# “Just to Jog My Memory”: An Examination of Forensic Interviewers’ Note-taking Behaviors and Perceptions of Notes With Child Witnesses

**DOI:** 10.1177/08862605241243346

**Published:** 2024-04-16

**Authors:** Shanna Williams, Kelly McWilliams

**Affiliations:** 1 McGill University, Montreal, QC, Canada; 2 CUNY, New York, NY, USA

**Keywords:** child sexual abuse, disclosure, forensic interviewing, maltreatment

## Abstract

In the current study, we surveyed forensic interviewers (*N* = 137) on their note-taking practices, perceptions of note-taking, and note-taking training. Many forensic interviewers surveyed (81%) reported that they take notes during forensic interviews. Of those, the most common reason for note-taking was to assist with remembering what the interviewee reported during the interview (89%) and to guide the formulation of follow-up questions (87%). Note-taking style was also reported upon, with most respondents indicating that they write down keywords that may be used again in the interview (78%), as well as short utterances or sentences related to the presenting narrative (61%). Finally, the majority (50%) of respondents who take notes reported always taking notes, although 29% reported taking notes most of the time. Of those respondents who reported not taking notes during forensic interviews, the majority listed the reasons as being that it distracts the child from the interview (85%) and causes them to break eye contact with the child (46%). Overall, many respondents endorsed the benefits of note-taking to the interviewing process, whereas a small minority reported some perceived risks or concerns with note-taking during interviews. Perhaps most notably, forensic interviewers, both of whom take notes and those who do not, reported low rates of note-taking training and a desire for more information on note-taking practices within the field. These results underscore the need for further research and best practice guidelines regarding note-taking during forensic interviews.

The role of the child witness in cases of maltreatment is important and unique (Hartman et al., 2023; Lyon et al., 2019), particularly given they are frequently the only person who can provide evidence of a crime (Finkel & DeJong, 1994; Smith et al., 2018). As a result of the difficulties in interviewing these vulnerable witnesses, standardized interviewing protocols have been developed to support child witnesses and their disclosures. Despite the efficacy of investigative interviewing protocols, field research has found that even when interviewers are trained on a protocol, adherence to best practices (e.g., use of open-ended questions) varies amongst professionals. While interviewers often grasp the big picture of the recommended strategies, they frequently struggle with the nuances of questioning children (Henderson et al., 2020), particularly in the formulation of high-quality invitation and cued invitation questions (Hershkowitz, 2001). However, there has been limited empirical work that has focused on additions to the forensic interview, which may support adherence to recommended practices. In the current empirical research, we investigated one potential support tool—the use of note-taking by forensic interviewers during a forensic interview.

## Tasks of Forensic Interviewing

Conducting forensic interviews with children is a cognitively demanding task. Often, forensic interviewers listen to children describe emotional and potentially upsetting instances of abuse while concurrently attending to the child’s narrative and adhering to a protocol of questioning. Adherence to recommended best practices, such as the use of invitations and cued invitations, requires interviewers to complete two tasks simultaneously. First, interviewers must attend to the child’s responses to process the broad meaning of the child’s words and construct logical follow-up questions. Additionally, interviewers must pay attention to the child’s language so that they can use the child’s words in their follow-up questions (i.e., cued invitations). In theory, note-taking could aid with the cognitive burden for each of these tasks, making it a practical and useful tool while interviewing. Note-taking may assist by creating a secondary source for interviewers to keep track of a child’s words and note keywords that require follow-up questioning (Baker et al., 2021).

Note-taking allows an interviewer to externally record keywords or entire topics that they will use to create follow-up questions. This means the interviewer can keep track of the important information a child says while not holding these concepts in memory. Support for this theory can be found in the education note-taking literature with the concept of the external storage effect (i.e., ESE; Di Vesta & Gray, 1972). According to ESE, note-taking during lectures affects intrinsic cognitive load (effort associated with a topic), a component of cognitive load theory (Costley et al., 2021). It is possible that if note-taking is completed to record keywords, this practice lessens the cognitive load demands on the interviewer, thus freeing up these mental efforts to be used in the formulation of invitation and cued-invitation questions. However, it is unknown if forensic interviewers follow this strategy when taking notes or if they take notes during forensic interviews in a different means, perhaps one that does not support protocol adherence (i.e., writing verbatim everything a child reports in the interview).

## Interviewers’ Perceptions of Note-taking

Although note-taking can be useful within the context of a forensic interview for the reasons stated, the benefits of this practice have been largely ignored by researchers in the fields of psychology and law. However, the practice of note-taking has been explored in other contexts where children are asked questions, such as therapy, and there are varying perceptions within the field. Notably, some practitioners have argued that note-taking should be discouraged when interviewing children because it could impact on rapport (Hickling et al., 1984; Miller, 1992; Mills, 2012; National Child Advocacy Center, 2016). Interviewers who take notes may be perceived as being less attentive to the interviewee or non-verbal communication by the interviewee may be missed by the interviewer (National Child Advocacy Center, 2016). Whereas some studies have reported an effect of note-taking on rapport (Hartley, 2002; Hickling et al., 1984; Miller, 1992), others have not found note-taking to influence rapport status in an interview (Christie et al., 2015). Those who have argued against note-taking in relation to rapport have argued that pauses for note-taking can impact the flow of the interview (Goldbloom, 2011). Conversely, Owens et al. (2010) and Sattler (2014) postulated the pauses needed for note-taking provide useful breaks for reflection. However, many of these concerns are found within the clinical psychology and medical literature rather than within the forensic interviewing literature (Lo & Wadsworth, 2014; Mills, 2012). Overall, there is a lack of consistency and information about note-taking, which may be perpetuated by a lack of empirical evidence supporting or discouraging the practice.

Similarly, social scientists have provided opinions and research examining the use of note-taking in various clinical practices. Hickling et al. (1984) found that therapists who took notes during sessions were rated significantly less effective than their counterparts who did not take notes during therapy sessions. However, other researchers have argued against this interpretation of note-taking during sessions. For example, when undergraduate students’ perceptions of school counselors who took notes versus those who did not take notes were surveyed, students did not report any differences between their perceptions of the two groups (i.e., those who took notes versus those who did not take notes) in terms of trustworthiness or competency (Miller, 1992). Additionally, Christie et al. (2015) examined the impact of note-taking on clients’ perceptions of a therapist and found no perceptions of distraction by respondents (Hickling et al., 1984; Miller, 1992; Mills, 2012). However, to date, we do not know how forensic interviewers perceive the act of note-taking and whether these perceptions mitigate or influence their note-taking during an interview.

Another issue to consider with note-taking, specific to the forensic context, is the potential legal consequences of documenting additional information during an interview (Baker et al., 2021). Forensic interviewers may not want to take notes during forensic interviewers out of fear that they would be used in court against a child witness. This concern has been noted in commentaries on note-taking practices during forensic interviews by researchers (e.g., Cauchi & Powell, 2009; MacFarlane, 1985; Vieth, 2009) and emerges from the rules of discovery, which dictate that prosecutors must turn over evidence in their possession to the defense if that evidence is exculpatory (i.e., points to the defendant’s innocence) and material to the outcome of the case (Brady v. Maryland, 1963). Understanding forensic interviewers’ concerns and perceptions of note-taking benefits will provide the groundwork for further research in testing these perceptions.

## Use of Note-taking by Forensic Interviewers

There has been a handful of studies that have examined the utility of notes in forensic interviews. Lamb et al. (2000) examined 20 forensic interviews of alleged sexual abuse victims and compared the notes of these interviews with the audio recordings of the same interviews. They report that when comparing handwritten notes to audio recordings of the interviews, forensic interviewers’ notes were not as accurate as an audio recording. The notes of the interviewers misrepresented the information provided by the child witness and they often did not attribute the correct questions to the elicited information. This research highlights a previous and continued concern within the research field: the need to record children’s statements in a forensic interviewing context to preserve the child’s statements and correctly submit their statements as evidence in court. Similarly, Powell et al. (2011) examined the effect of instructing forensic interviewers on note-taking strategies and the impact on the completeness of their notes. Overall findings suggest that if provided strategies the completeness and accuracy of forensic interviewers’ notes increased. While the above research provides important information regarding the need to record interviews, the findings regarding note-taking only apply to note-taking as a documentation tool, not as a support tool. In other words, there is a need to examine note-taking from the lens of supporting interviewers to see if there are potential benefits.

## Current Study

To our knowledge, there is no published research examining forensic interviewers’ note-taking behaviors and perceptions of note-taking during forensic interviews. Although researchers have examined the quality of notes taken during forensic interviews (Lamb et al., 2000; Powell et al., 2011) no one has focused their attention on interviewers’ note-taking behaviors or their perceptions of note-taking generally. The current study had several aims. First, we sought to explore forensic interviewers’ note-taking behaviors (e.g., whether they take notes, how often, and what style they use; Aim (1). Next, we sought to examine forensic interviewers’ perceptions of the benefits and risks of note-taking in interviews with children (Aim 2). We examined forensic interviewers’ note-taking behaviors by asking respondents of a survey a series of close-ended and open-ended questions on whether they take notes, reasons they do or do not take notes, the style of their note-taking, and the frequency of note-taking. As part of this aim, we also examined note-taking perceptions by asking participants to provide a series of true/false responses about note-taking. We specifically examined respondents’ perceptions of note-taking about their experience with forensic interviewing (i.e., years of experience in the field).

Given the lack of previous research on the subject matter, we did not have directional hypotheses for many of the questions in the survey. Instead, our hypotheses were exploratory in nature. However, based on the clinical and medical literature that is available we had the following prediction. Previous research has noted that documentation of what witnesses say should not be done through notes rather through recording. Therefore, we predict that interviewers will not use notes to document what is being disclosed in interviews.

## Method

### Participants and Design

We identified all Children and Youth Advocacy Centers (CYACs) in Canada and a portion of Child Advocacy Centers (CACs) in the United States that provide forensic interviews for children who have alleged maltreatment. Research assistants sent out emails to the identified list of 30 CYACs in Canada and 352 CACs in the United States. We also posted our survey to the data collection website Prolific, with survey questions embedded at the beginning of the survey to identify participants who conduct forensic interviews. A total of 191 respondents completed the survey; however, 11 were removed from the sample because they completed <30% of the survey. Of the remaining 180, 8 were recruited through the prolific platform and 172 through email recruitment. Using the strategy of the previous empirical paper with samples from CYAC/CACs (Cauchi & Powell, 2009), we calculated our response rate by numerating any participant response from a CYAC/CAC as a response from that organization. Overall, we sent recruitment emails to 478 CYAC/CACs across Canada and the United States and received at least one response from 102 of those organizations. Counting each response from one organization across all organizations contacted for the survey resulted in a response rate of 21.34% for those responses from the email recruitment. For prolific, the response rate was unknown.

### Materials

The survey was developed by the authors and based on previous research examining forensic interviewers’ preparation methods (i.e., Rivard & Compo, 2017), note-taking practices in clinical interviews (Hickling et al., 1984) and previous research examining note-taking in forensic interviewing contexts (Lamb et al., 2000). The questionnaire contained 105 items and was broken into three sections: (a) employment and experience, (b) note-taking behaviors, and (c) perceptions of note-taking (See Open Science Framework: https://osf.io/sjh56/?view_only=58d336de71d54c10be3d1d00022123e7). Each section contained both open-ended and close-ended questions. For all questions, participants were provided with an option to not respond, and as a result, the total number of responses varied throughout the questionnaire. Following the conclusion of the survey, participants were thanked for their participation in the study and invited to provide their email addresses to receive results of the study.

### Materials and Procedure

The survey was approved for distribution by the authors’ university ethics committee (Blind for review) and was hosted on the online survey software Qualtrics. The survey took an estimated 20 to 25 min to complete.

### Coding Scheme

Many questions in the survey were quantitative; however, a portion were qualitative, and as a result, thematic analysis was conducted for all open-ended responses. The analysis was inductive in process, whereby the two coders read all open-ended responses independently and created major themes related to the responses (Braun & Clarke, 2006). The coders met to review the potential themes and subthemes, as well as to define the coding criteria for each theme. The coders then separately completed the coding of all open-ended responses with the agreed upon thematic framework and compared their response agreement. The coding was done by the authors, and therefore they were not blind to the study goals. However, there were no directional hypotheses regarding the open-ended responses.

### Inter-rater Reliability

Two coders independently coded 100% of responses and reached acceptable levels of inter-rater reliability (Kappa > .78 on all codes). The coders were not blind to the hypotheses of the research during the development of themes and coding. Any discrepancies in coding were resolved by the authors and assigned by the principal coder for analysis.

### Statistical Power

A power analysis indicated a sample of at least 128 participants would be needed in order to detect an effect size of 0.25 (i.e., medium effect size), with an alpha level of .05 and power of .80. Our study included a final sample of 137 participants, which is slightly greater than what the power analysis indicates would be necessary. Therefore, our analysis is adequately powered.

## Results

A total of 191 participants responded to the survey. We excluded participants for three reasons. First, 11 participants were removed because they completed less than 30% of the survey. Second, 25 respondents were excluded because they reported not conducting forensic interviews as part of their employment. Lastly, we removed 18 participants who indicated that they did not want to respond to the questions regarding note-taking during forensic interviews or failed to respond to questions indicating their note-taking practices. The final sample included 137 participants; of these respondents, 91% (*n* = 125) self-reported as Female. Seventy-eight percent (*n* = 107) reported residing in the United States, whereas 22% (*n* = 30) reported being located in Canada. Eighty-three percent (*n* = 114) of respondents were Non-Hispanic White, 6% (*n* = 8) were Hispanic/Latine, 6% (*n* = 8) Black, 3% (*n* = 5) Native American/Indigenous, and 1% (*n* = 2) Asian/Pacific Islander.

### Employment and Experience

The full sample included professionals from various domains. Forty-two percent (*n* = 57) of the sample self-reported their profession as social worker, 41% (*n* = 56) identified as forensic interviewers, 12% (*n* = 17) were police officers, 4% (*n* = 5) were clinical providers (psychologist, therapist, or psychotherapist), and two participants were attorneys (one defense attorney and one prosecutor). The average years of experience reported was 11.27 (*SD* = 8.21), with a range of 1 to 40 years and a median of 10 years of experience. The majority of participants reported that they conduct interviews to investigate child maltreatment (99%, *n* = 136, one respondent did not respond), all 136 participants reported conducting interviews for physical abuse and sexual abuse, and 93% (*n* = 127) said they interview children about suspected neglect. In terms of frequency of interviewing, 33% (*n* = 45) of respondents reported completing interviews multiple times a day, 41% (*n* = 56) reported interviewing daily, 6% (*n* = 8) reported weekly, 8% (*n* = 11) reported biweekly, 4% (*n* = 6) reported monthly, 7% (*n* = 9) reported quarterly, and 1 person preferred not to respond.

A significant majority of our sample had received formal training on how to conduct forensic interviews with children and youth (99%, *n* = 135); however, only 49% (*n* = 47) of the sample reported that they had received training/instruction on how to take notes when interviewing children.

### Note-taking Behavior

To address our first aim, exploring note-taking behavior, we examined whether or not participants endorse note-taking generally. We asked respondents whether they take notes during forensic interviews with children and found that 81% (*n* = 111) of participants reported taking notes during forensic interviews. Using their response to whether they take notes, we split our sample into two note-taking groups: (1) note-takers and (2) non-notetakers. Given the aim to explore the note-taking behaviors, it is likely variables are different for those who choose to take notes versus those who choose not to take notes, therefore, we report their responses separately. Because we split our participants into these note-taking groups, we conducted analyses to examine whether the groups differed significantly by country of origin, years of experience, and/or note-taking training. No significant differences between groups were observed for these variables. For country of origin (*X*2 [1, 137] = 2.01, *p* = .16), participants from Canada and the United States were distributed similarly across the note-taking (CA: 90%, *n* = 27, U.S.: 78%, *n* = 84) and non-note-taking groups (CA:10%, *n* = 3, U.S.: 22%, *n* = 23). The average years of experience in both groups was similar (*t* [89] = 1.38, *p* = .17); those who reported taking notes had an average of 11.19 years of experience (*SD* = 7.99), and those who do not take notes had been in their profession for an average of 14.08 years (*SD* = 7.79). Finally, there was no significant difference reported in note-taking training by group (*X*2 (1, 136) = 2.16, *p* = .14). Fifty-two percent (*n* = 58) of the note-taking group and 36% (*n* = 9) of the non-note-taking group reported they had received some form of training on note-taking.

Next, we examined specific note-taking behaviors by asking participants about (a) their reasons for taking notes, (b) their style of note-taking, and (c) their frequency of note-taking.

### Reasons for Taking Notes

#### Note-takers

For participants who indicated they do take notes during forensic interviews (*n* = 111), we asked them to select from a list of reasons why they chose to do so. The most common reasons were to assist with remembering what the interviewee reported during the interview (89%) and to guide the interviewers’ follow-up questions (87%). A moderate number of respondents reported using note-taking to assist with engagement in the interview (41%) and with filling out reports following the interview (32%). A smaller minority of respondents reported notes as a record for the court (16%), to allow for pauses for the interviewee (15%) or interviewer (11%), or for other reasons not stated (3%)

In addition to choosing options from a list, we also asked note-takers an open-ended question about why they take notes. Although many of the participants’ open-ended responses described reasons captured in their closed-ended responses, a few reasons beyond what was provided in the list emerged. The themes in their responses could be grouped into four areas: (1) accuracy/remembering details (e.g., “to remember language child used”, “to remember key names and dates”; “to keep track of information”); (2) to refer back for questioning (e.g., “remember key points to ask follow-up questions of without interrupting child’s narrative”); (3) impression management (e.g., to stay present in the interview and to show the child the forensic interviewer is taking their disclosure seriously); and (4) timelines/multiple incidents (e.g., “noting the timeline of incidents if more than one”; “. . .so I can remember chronologically what happened”).

#### Non-Notetakers

For those respondents who reported they do not take notes during forensic interviews (*n* = 26), we asked respondents to select from a list of reasons why they do not take notes. The most popular reasons were that interviewers were concerned note-taking removes them from interaction with the child (85%), distracts them from the interview (50%), causes them to break eye contact with the child (46%), and they feel they can remember enough on their own without notes (42%). Less common reasons were that their supervisor or center has a policy against note-taking (15%), the interviewers do not think notes help (8%), or other (3%).

When asked an open-ended question on why they do not take notes, participants’ responses indicated themes that were consistent with what was stated in a close-ended question. The most common was that they felt note-taking would be distracting (i.e., “interfere with rapport”; “distracting,” “disengaging”). Other reasons included: (a) someone else takes the notes (e.g., “I have a monitor completing the task for me”; . . .MDT members watching live, so I can be fully in the conversation with the child while they listen and take notes”), (b) that the notes would be a liability for court cases (e.g., “I don’t want them to become an issue if the case goes to court”) or (c) not permitted (e.g., “My director does not allow note-taking but I wish I could take notes sometimes.).

### Style of Note-taking

We asked participants who do take notes (*n* = 111) to report on their note-taking style. We asked respondents to choose from a list of statements that were consistent with the format they used for taking notes. The most common strategy endorsed was writing down keywords that will be used again in the interview (78%), followed closely by writing down utterances or sentences related to the presenting concern (61%). A moderate number of participants reported that they use their notes to attempt to summarize the gist of what the child is telling them (25%). Small numbers reported that they write information in their notes for a follow-up interview (7%), attempt to write down every word the interviewee says during the forensic interview (6%), or write impressions not related to what they will ask the child (5%).

Respondents were also asked to provide an open-ended response on their note-taking methods (i.e., describe your note-taking methods). Overall, responses could be separated into the following main themes: (a) how interviewers took notes (e.g., notepads, pencil/pen, handwritten, bullet points), (b) information-driven format (e.g., name and demographic information); (c) specific layout, and (d) report or court-related concerns (e.g., using notes to write a report for their agency). Several respondents reported using paper and pencil or some handwritten format for their note-taking methods. Information-driven responses reported upon the notes being to assist with salient details such as the child’s name, names of events, or dates. Of those who reported upon a specific layout, many reported using visual organization strategies for their notes, such as diagrams organizing their notes page in a specific format. Of those who reported upon specific court concerns, some respondents reported omitting information from notes out of concern for legal issues (i.e., omission of names) or using notes to assist with later report writing required for their agencies.

### Frequency of Note-taking

The final note-taking behavior we examined was how often participants take notes. Overall, of the participants who take notes (*n* = 111), 50% of respondents reported always taking notes during forensic interviews, while 29% reported taking notes most of the time. Six percent of respondents reported taking notes half of the time, 14% reported sometimes taking notes, and one participant did not respond regarding frequency.

### Perceptions of Note-taking

The second aim of our survey was to measure interviewers’ perceptions of the benefits and risks of note-taking. To measure this, we asked respondents to reply to a series of true/false statements regarding positive and negative statements about note-taking during forensic interviews (Table 1). A total of 104 of the participants responded to this portion of the survey; 33 participants did not complete this portion. Of the 104 who completed this portion, 87 participants were note-takers, and 17 were non-notetakers. First, we examine their overall perceptions, then because it is possible that perceptions of note-taking benefits and risks could be influenced by employment experience; we also examined whether participants’ endorsements were related to their years of experience or training. To do this we calculated the percent of “true” responses each participant endorsed for benefits (i.e., sum/3) and for risks (i.e., sum/6). For note-takers, we conducted two separate analyses to examine relations to years of experience and training. We examined the relation to years of experience using bivariate correlations, and we examined the relation to training using one-way analyses of variance (ANOVAs). These results are reported in the sections below. Unfortunately, the number of “non-notetakers” was too low to run meaningful inferential statistical tests, therefore, we examined the relation with employment history descriptively. To examine the relation to years’ experience, we performed a mean split based on the average amount of years in the profession for non-notetakers (*M* = 14.08, *SD* = 7.78) and reported the average endorsement for those who worked shorter than the average amount of time (*n* = 10) and those who have worked longer than average amount of time (*n* = 7). For training, we simply report the average endorsements for those who have and have not received note-taking training.

**Table 1.** Perceptions of the Benefits and Risks of Note-taking by Note-taking Groups.

Benefits	Note-takers (*n* = 87)			Non-notetakers (*n* = 17)		
	True	False	Unsure	True	False	Unsure
Note-taking aids in my recall of what occurred during the interview.	0.92	0.01	0.07	0.53	0.29	0.18
Note-taking helps me stick to my training during an interview.	0.63	0.17	0.20	0.12	0.59	0.29
Note-taking allows for breaks/pauses that provide time for reflection.	0.67	0.08	0.25	0.35	0.35	0.29
AVERAGE	0.74	0.09	0.17	0.33	0.41	0.25
	Note-takers (*n* = 87)			Non-notetakers (*n* = 17)		
Risks	True	False	Unsure	True	False	Unsure
Note-taking hinders the rapport and connection I have during an interview.	0.16	0.69	0.15	0.77	0.12	0.12
Note-taking is distracting to me when I am engaging in an interview.	0.15	0.75	0.10	0.77	0.18	0.06
Many interviewees find the act of note-taking during an interview off-putting or suspicious.	0.28	0.40	0.32	0.53	0.06	0.41
Note-taking makes it harder for me to stick to my training during an interview.	0.06	0.84	0.10	0.29	0.29	0.42
I often feel that if I take notes, I miss things during an interview.	0.16	0.72	0.12	0.71	0.12	0.18
Note-taking can make the tone of an interview seem more formal than I would like it to be.	0.31	0.53	0.16	0.77	0.23	0.00
Average	0.17	0.66	.16	0.64	0.17	0.20

### Benefits

#### Note-takers

Among notetakers, there is a consensus about the benefits of note-taking. Across all three individual statements about the benefits of note-taking, most responses indicated an endorsement of note-taking benefits (74%), a moderate number of responses were unsure, suggesting some insecurity about the stated benefits (17%), and a small number of responses explicitly refuted the benefits (9%). When we examined responses for each specific belief, we found that the most endorsed benefit was that note-taking aids in recall of what occurred during the interview (92% true responses). The least commonly endorsed benefit was that note-taking helps interviewers stick to their training (63% true responses) of participants believed to be true.

When we examined whether endorsements of benefits were related to employment experience, we found that years of experience were significantly negatively correlated with participants’ belief in the benefits of note-taking (*r* = −.27, *p* = .04). The more senior interviewers endorsed fewer note-taking benefits than those who had been interviewing for less time. However, endorsement of the benefits of note-taking did not differ by whether the participant had been trained in note-taking (*F* (1, 86) = .66, *p* = .80, η2 = .001). Participants who had note-taking training (*M* = .75, *SD* = .30) and who had no note-taking training (*M* = .74, *SD* = .31) endorsed the benefits of note-taking at similar rates.

#### Non-notetakers

Non-notetakers’ beliefs on the benefits of note-taking were mixed. More specifically, only 33% of responses indicated that non-notetakers believed the benefits of note-taking to be true, whereas 41% of responses believed the benefits to be false, and 25% of responses indicated that non-notetakers were unsure about the stated benefits. The most endorsed benefit among non-notetakers (like notetakers) was that note-taking can aid in their recall of what occurred during the interview (53% true responses). The benefit that the fewest number of participants supported was that note-taking helps them stick to their training (12% true responses).

When we look at their responses in relation to employment history descriptively, there did not seem to be a visible relation to non-notetakers’ perception of the benefits of note-taking. For years’ experience, those who have worked less than the average amount of time for non-notetakers endorsed the benefits (*M* = .33, *SD* = .40) at a similarly low rate to those who have been working as an interview longer than average for non-notetakers (*n* = 7; *M* = .20, *SD* = .30). Training also does not seem to make a difference, descriptively, as those who received training (*M* = .33, *SD* = .40) and those who did not (*M* = .25, *SD* = .30) both endorsed few benefits of note-taking.

### Risks

#### Note-takers

On average, note-takers did not endorse the provided risks of note-taking. Across the statements about risk, only 17% of the responses indicated support for the stated risks, whereas 66% of the responses denied a belief in the risks. Sixteen percent of participants indicated that they were unsure of the risks of note-taking. When we examined the responses for specific statements, we found the most common concern among the note-taking participants was that note-taking could make the tone of the interview seem more formal than it needs to be (31% true responses). The least common concern was note-taking makes it hard for the participant to stick to their training (6%).

Employment history was not significantly related to note-takers’ perceptions of the risks of note-taking. Specifically, years of experience was not significantly correlated to the percentage of risks endorsed by note-takers (*r* = −.03, *p* = .84), nor was training associated with percentage of risk endorsements (*F* (1, 86) = 1.15, *p* = .29, η^2^ = .01). Those with (*M* = .22, *SD* = .30) and without training (*M* = .16, *SD* = .28) did not endorse notetaking risks very often.

#### Non-notetakers

On average, non-notetakers did support the perceived risks of note-taking. Across the statements about risk, 64% of the responses endorsed the risks of note-taking, 17% reflected they did not believe the risk of note-taking, and 20% indicated that they were unsure of the risks of note-taking. There were three specific risks that were equally endorsed among the non-notetakers: (1) note-taking hinders rapport and connection during the interview, (2) note-taking is distracting when engaging in an interview, and (3) note-taking can make the tone of the interview more formal than it needs to be (all 77%). The least common concern among the non-notetakers (as it was for the notetakers) was that note-taking makes it harder for them to stick to their training (29%).

Again, employment history did not seem to play a role in non-notetakers perceptions. Those with longer than average (*M* = .66, *SD* = .20) and shorter than average (*M* = .63, *SD* = .20) years’ experience endorsed, on average, about 60% of the risks of note-taking. Endorsement rates across training experience were also similar; non-notetakers who had note-taking training had an average endorsement rate of 67% (*SD* = .33), and non-notetakers who had no note-taking training had an average endorsement rate of 69% (*SD* = .17).

## Discussion

This study is the first empirical research to document forensic interviewers’ perceptions of note-taking and one of only a few studies (Baker et al., 2021; Lamb et al., 2000) to examine interviewers’ self-reported note-taking behaviors. We had two primary aims: the first was to examine the frequency of note-taking during forensic interviews, and the second was to examine forensic interviewers’ perceptions of note-taking. To examine these two aims we designed and administered an internet-based survey across both the United States and Canada. Responses (*N* forensic interviewers = 137) resulted in several notable findings related to forensic interviewers’ note-taking behaviors as well as their perceptions of notes during interviews.

First, the majority of forensic interviewers in our survey reported taking notes during forensic interviews. This is an important finding as it indicates that note-taking is actively being used during forensic interviews. Furthermore, most of these interviewers indicated taking notes to remember what child witnesses were reporting and to assist with follow-up questioning. This supports the argument that note-taking may be used as a tool for interviewers rather than as just a method of documentation, which previous research has failed to examine as a purpose for note-taking (Baker et al., 2021). Notably, those respondents who did not report taking notes during forensic interviews endorsed reasons that are frequently cited within the literature (Hickling et al., 1984; Miller, 1992; Mills, 2012; National Child Advocacy Center, 2016) such as distractions or interference with rapport.

Second, we aimed to examine perceptions of note-taking by both those who take notes and those who do not take notes. The majority of those who take notes during forensic interviews reported favorable perceptions of note-taking. Specifically, most respondents who took notes endorsed favorable perceptions of notetaking. When asked to identify what aspects of note-taking were favorable most respondents reported aiding in recall of what occurred in the interview, and noted adherence to training as a benefit. Notably, many trainee resources feature only a small section about note-taking (Sternberg et al., 2001), if it is included at all. Moreover, recommendations for the practice of note-taking in such resources often lack an empirical basis (Groth-Marnat & Wright, 2016; Owens et al., 2010; Sattler, 2014). The small number of studies that have examined note-taking within forensic interviews have largely focused on the failures of note-taking as a documentation strategy (Cauchi & Powell, 2009; Lamb et al., 2000) rather than the potential benefits as a support tool (Baker et al., 2021). As a result, the current findings are important and highlight a gap in the current literature.

Of those who reported not taking notes, the majority endorsed some perceived risks with note-taking and most of these respondents endorsed risks such as rapport building and note-taking as a distracting act to the child interviewee. Additionally, these respondents also reported note-taking, changing the tone of interviews to making them appear more formal in nature. Non-notetakers did not appear to note legal concerns as a direct reason to omit note-taking practices from forensic interviews. However, the concern of court and discovery was a concern for those who did take notes and appeared to influence how and what information forensic interviewers may include in their notes.

In terms of perceptions of note-taking, another additional finding was years of experience had a negative relation to perceptions of note-taking during interviews. Specifically, those with more experience endorsed fewer benefits to note-taking compared to those more novice interviewers. It may be that those with less experience interviewing used note-taking as a tool to assist with adherence to best practice guidelines and their training compared to more experienced interviewers who felt more confident in their adherence or approaches.

One additional surprising and important finding from the survey was the lack of training and desire for more formal instruction on how to take notes. Given most of the respondents reported taking notes in some form during forensic interviews it was interesting that many respondents indicated a desire for further training and instruction on note-taking. Notably, only 52% of the note-taking group and 36% of the non-note-taking group reported any form of training on note-taking. The current results support the argument that forensic interviewers may benefit from more specific training on note-taking.

### Limitations and Future Directions

While the findings of this study are novel and address a gap in the literature, there are limitations that need to be addressed in future research. First, this study relied on forensic interviews to self-select into the study, which means there is a potential for response bias, in that those forensic interviewers who take notes may have been more likely to take an interest in the survey and thus agree to participate. An alternate and future direction would be to systematically observe forensic interviews within specific locations (i.e., CYAC) for note-taking behaviors of interviewers. Second, although our sample was diverse in terms of collecting from both Canada and the United States, a larger sample representative of all CYAC/CACs may help provide more generalizability to the current findings. Moreover, although a number of different geographic locations were surveyed (i.e., respondents indicated several states and provinces of response) it is possible that certain agencies were overrepresented within the sample. As a result, it would be helpful in future studies to examine which agencies respondents worked for and whether a specific CAC/CYAC was overly represented within the data set. Third, we collected self-report data, meaning that our results represent interviewers’ perceptions both of note-taking generally and also their perceptions of their note-taking behavior, which may not accurately reflect their actual behaviors. Future research will need to include observational work to determine if interviewers’ perceptions of their note-taking behaviors actually align with their true actions during interviews. Fourth, regarding coding of the open-ended responses, coders were not blind to predictions based on the open-ended responses. Although there was a small number of questions being addressed by the qualitative analysis of the research, there was potential for bias as a result of the coders not being blind to the hypotheses of the research, and certain themes could have been missed. Future research should examine qualitative responses with coders who are blind to the overall aims and predictions of the research. Fifth, the current study provides mostly descriptive data. Future work should more closely examine potential group differences that may drive differences in note-taking behaviors. Finally, next steps need to examine the efficacy of different styles of note-taking. The current findings provide information regarding what type of notes interviewers are taking; future research needs to experimentally examine which techniques are associated with adherence to recommended best practices. Such experimental work could help support the development of specific guidelines for forensic interviewers.

## Conclusion

Forensic interviewers appear to be using notes, although not for the reasons previously examined in the literature. Rather than note-taking being for the purpose of documenting by the forensic interviewers, forensic interviewers are using notes to assist with adherence to best practice protocols. Moving forward, better support and training should be developed to address the use of note-taking in forensic interviews.
